# Compound-specific radiocarbon dating and mitochondrial DNA analysis of the Pleistocene hominin from Salkhit Mongolia

**DOI:** 10.1038/s41467-018-08018-8

**Published:** 2019-01-30

**Authors:** Thibaut Devièse, Diyendo Massilani, Seonbok Yi, Daniel Comeskey, Sarah Nagel, Birgit Nickel, Erika Ribechini, Jungeun Lee, Damdinsuren Tseveendorj, Byambaa Gunchinsuren, Matthias Meyer, Svante Pääbo, Tom Higham

**Affiliations:** 1 Oxford Radiocarbon Accelerator Unit, Research Laboratory for Archaeology and the History of Art, University of Oxford, Dyson Perrins Building, South Parks Road, Oxford, OX1 3QY UK; 2 Max-Planck-Institute for Evolutionary Anthropology, Deutscher Platz 6, D-04103 Leipzig, Germany; 3 Seoul National University, Gwanak-ro 1, Gwanak-gu, Seoul, 08826 Korea; 4 Dipartimento di Chimica e Chimica Industriale, Università di Pisa, Via G. Moruzzi 13, Pisa, 56124 Italy; 5 Institute of History and Archaeology, Mongolian Academy of Sciences, Jucov St 77, Ulaanbaatar, 13343 Mongolia

## Abstract

A skullcap found in the Salkhit Valley in northeast Mongolia is, to our knowledge, the only Pleistocene hominin fossil found in the country. It was initially described as an individual with possible archaic affinities, but its ancestry has been debated since the discovery. Here, we determine the age of the Salkhit skull by compound-specific radiocarbon dating of hydroxyproline to 34,950–33,900 Cal. BP (at 95% probability), placing the Salkhit individual in the Early Upper Paleolithic period. We reconstruct the complete mitochondrial genome (mtDNA) of the specimen. It falls within a group of modern human mtDNAs (haplogroup N) that is widespread in Eurasia today. The results now place the specimen into its proper chronometric and biological context and allow us to begin integrating it with other evidence for the human occupation of this region during the Paleolithic, as well as wider Pleistocene sequences across Eurasia.

## Abstract (web summary)

The Salkhit skull from Mongolia was initially suggested to have archaic hominin characters. Here, Devièse and colleagues date the skull to approximately 34–35 thousand years ago and reconstruct its mitochondrial genome, finding that it falls within modern human haplogroup N found across Eurasia.

## Introduction

**Fig. 1:**
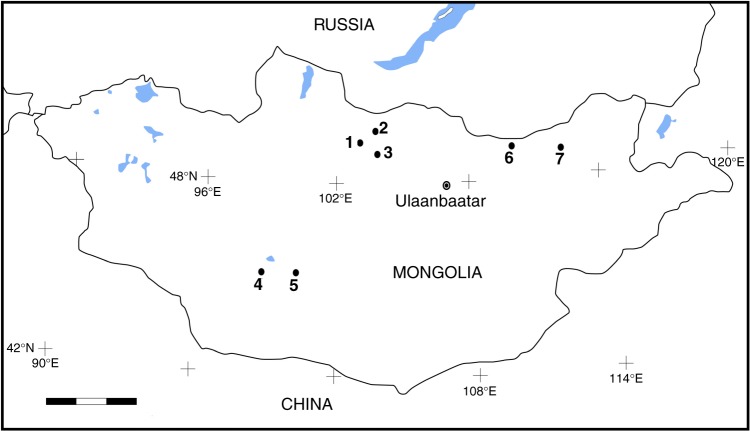
Map showing Upper Paleolithic sites in Mongolia. 1—Tolbor sites, 2—Dörölj, 3—Orkhon sites, 4—Chickhen sites, 5—Tsagaan Agui, 6—Rashaan Khad and 7—Salkhit. Chronometric data for these sites is listed in Supplementary Table 1. Blocks on scale bars represent 100 km

Mongolia has been a focus of attention for researchers interested in human origins for more than a century and substantial advances in the understanding of the Mongolian Paleolithic have been made in the last few decades^1^. The discovery of Paleolithic remains is, however, still mainly limited to restricted areas in the centre part of the country (Fig. 1, Supplementary Table 1). In addition to surface surveys, recent excavations revealed aspects of the Paleolithic occupation of the region, enabling archaeologists to begin integrating the Mongolian Paleolithic with other Pleistocene sequences across Eurasia. In congruence with the neighbouring regions of southern Siberia and the Siberian Altai, the Upper Paleolithic in Mongolia has been divided into three phases: Early, Middle and Late Upper Paleolithic. In contrast with the Lower and Middle Paleolithic, which are characterized by few sites, the start of the Upper Paleolithic is relatively well defined by the presumably persistence of earlier Mousterian or Levallois industries and the appearance of blade production in Northern Mongolia, mirroring the southern Siberian record^2^. The Early Upper Paleolithic (EUP) is usually subdivided into two phases. The first, spanning from 40,000 BP to 35,000 BP, includes the site of Rashaan Khad, which is about 150 km SW from Salkhit (dated at 39,100 ± 1000 BP (OxA-34324))^3^. The second, represented by assemblages from the Khangai Mountains and the Gobi Altai district, covers the period between 33,000 BP and 26,000 BP^1,4,5^. The Mousterian or Levallois elements disappear with the Middle Upper Paleolithic (MUP) in Mongolia. MUP sites have been identified only in the Orkhon River valley and are younger than ca. 25,000 BP^1^. Finally, the later phase of the Mongolian Upper Paleolithic (LUP) is characterized by microliths and lasts until the end of the Pleistocene^1–3^.

The skullcap analysed in this study was discovered in 2006 during mining operations in the Salkhit Valley of the Norovlin county in the Khentii province, eastern Mongolia (48°16′17.9″ N and 112°21′37.9″ E). It is, so far, the only Pleistocene human fossil found in the country. Subsequent investigations around the find-spot did not yield additional fossil remains. The skullcap has a mostly complete frontal as well as two partially complete parietal and nasal bones. Ages suggested for the specimen range from the Early Middle Pleistocene to the terminal Late Pleistocene. The presence of archaic features has led to a potential affiliation to an uncharacterized archaic hominin species. The specimen was thus initially referred to as *Mongolanthropus*^6^. The comparison of the dimensions of the skullcap with reference skullcaps by multidimensional scaling analysis showed similarities with *Neanderthals*, *Homo erectus* and Asian archaic *Homo sapiens*^7^. Based on the published photographs, Kaifu and Fujita^8^ suggested that the Salkhit specimen belongs to a terminal Pleistocene modern human. Additional comparisons with Middle and Late Pleistocene hominin fossils from northeast Asia (Zhoukoudian Locality 1, Dali, and Zhoukoudian Upper Cave) concluded that the peculiar features of the Salkhit skull are more likely to be regionally predominant modern human features than diagnostic features of an archaic species^9,10^.

Because of the dearth of hominin fossils recovered in Mongolia, the Salkhit skull represents a unique opportunity to investigate the types of humans that occupied the region during the Pleistocene. Here, we present the results of both chronometric and genetic analyses of the Salkhit specimen. This fossil dates to approximately 34–35 thousand years ago and its mitochondrial genome is of Eurasian modern human type.

## Results and Discussion

### Fossil specimen

**Fig. 2:**
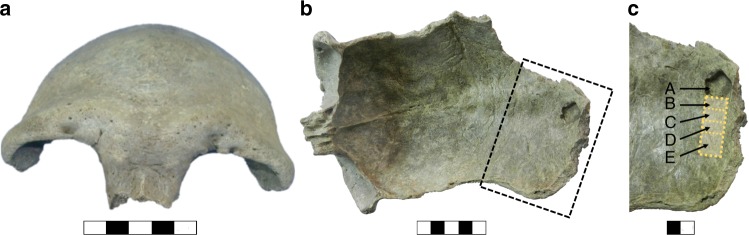
The Salkhit skullcap. In *norma frontalis* (1), in *norma basilaris* showing the first sampling for dating A (2) and the areas of sampling B, C, D and E (3) for genetic analyses and compound-specific radiocarbon dating. Blocks on scale bars represent 1 cm

Since its discovery in 2006, the Salkhit fossil (Reference number: 2006-4) has been kept at the Institute of History and Archaeology (formerly, Institute of Archaeology), Mongolian Academy of Sciences, in Ulaanbaatar. The specimen was sampled for radiocarbon and genetic analyses in four locations (B, C, D and E, Fig. 2) in the internal part of the skull at the posterior tip next to an area that had been sampled prior to this study (A, Fig. 2). In total, ~1 g of bone was sampled.

### AMS dating

The Salkhit skull (Sample A, Fig. 2) was previously dated to 22,105 ± 45 BP by the Beta Analytic Inc. radiocarbon laboratory (Miami, Florida, USA), but this result has never been formally reported^11^. Unfortunately, no information is available about the sample preparation methods that had been applied. Another fraction of the same sample A was also dated at the Oxford Radiocarbon Accelerator Unit (ORAU) in 2010. It was pre-treated following the routine procedure comprising decalcification, base wash, reacidification, gelatinisation and ultrafiltration steps (Coded ‘AF’ in the ORAU), as described by Brock et al.^12^. It produced a date of 23,630 ± 160 BP (Table 1). The C:N atomic ratio of carbon to nitrogen for the extracted collagen was 3.5. Even though this value falls within a range of C:N values [2.9–3.5] that passes the quality control criteria of most radiocarbon facilities, the value is relatively high and could indicate that some of the contamination was not entirely removed from the sample. We therefore decided to re-date the skullcap using a compound-specific approach^13^. The higher efficiency of this method in removing contaminants from heavily contaminated samples and its ability to provide accurate ^14^C measurements has been demonstrated in several recent cases involving Paleolithic sites in France^14^, Croatia^15^, Russia^16–21,^ and the Americas^22,23^.

**Fig. 3:**
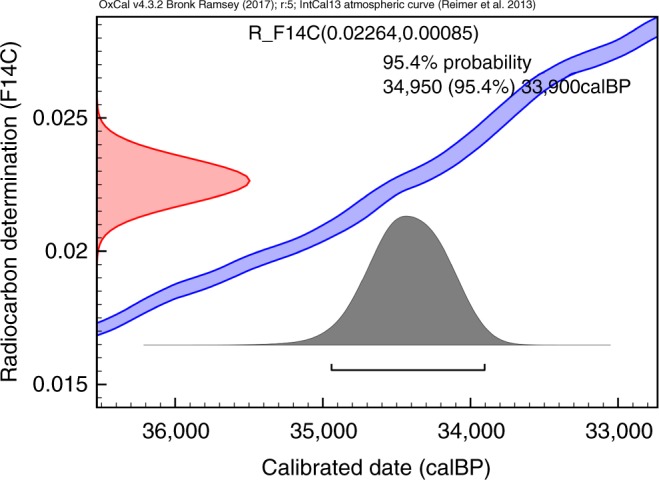
Calibration of the radiocarbon measurement. The left-hand axis shows the radiocarbon concentration (F^14^C or fraction modern) and the bottom axis shows the calibrated age obtained using the IntCal13 calibration curve of Reimer et al.^25^. Figure produced using OxCal^24^

**Fig. 4:**
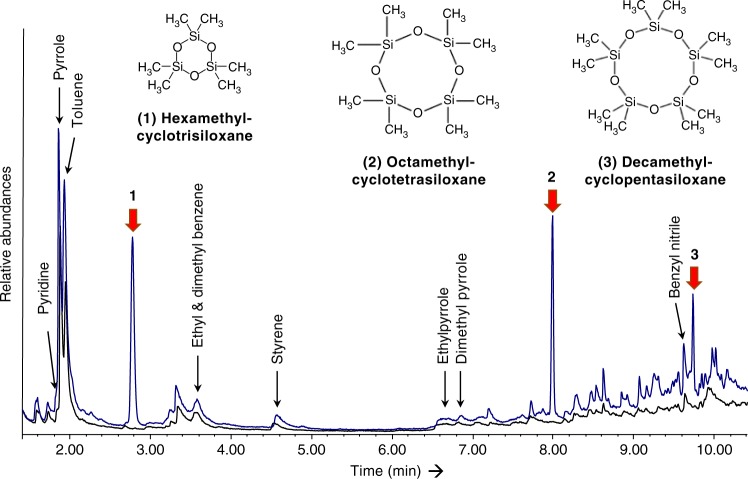
Py-GC/MS characterization. Blue profile—Pyrogram obtained on 450 µg of the Salkhit bone; Black profile—Pyrogram obtained on 220 µg of an uncontaminated bone (Latton, UK) used as a standard at the ORAU. The main compounds identified in both profiles are pyrolytic products of collagen^51,52^. The profile of the Salkhit sample also shows the presence of 3 compounds with siloxane structure (red arrows) that are reported as pyrolysis products of silicone^53^ leading to assess the residual presence of such material within the bone matrix

A new bone sample was obtained and after extraction, the collagen was hydrolyzed and the amino acid hydroxyproline was isolated using preparative High Performance Liquid Chromatography (Prep-HPLC). The hydroxyproline was combusted, graphitised, and measured by AMS, producing a date of 30,430 ± 300 BP (OxA-X-2717-25). The C:N atomic ratio measured was 5.0, corresponding to the expected theoretical value for hydroxyproline (C_5_H_9_NO_3_) and indicating the absence of contamination in the sample after the Prep-HPLC treatment. The date were calibrated using OxCal 4.3^24^ and the INTCAL13 calibration curve^25^, yielding an age range of 34,950–33,900 Cal BP (at 95% probability) (Table 1, Fig. 3). This is significantly older than the two dates obtained previously. Such differences have been observed before in cases where bones were heavily contaminated with conservation agents, indicating that the standard chemical pretreatment^12^ is sometimes not efficient enough to remove all high molecular-weight contamination that is present in a sample and sometimes even cross-linked to the collagen. The contamination was confirmed by additional chemical investigations using Pyrolysis Gas Chromatography coupled with Mass Spectrometry (Py-GC/MS). They revealed the presence of a silicone-based contaminant in the Salkhit bone probably used to make the endocast (Fig. 4)^7^. The hydrolysis of the collagen and isolation of the amino acid hydroxyproline obviated this problem.

**Table 1:** Radiocarbon dates of the Salkhit skull

Lab reference	Conventional ^14^C age (BP)	P Code	Bone Used (mg)	UF gelatin yield (mg)	%Yld	%C	δ^13^C (‰) (VPDB)	δ^15^N (‰) (AIR)	C/N
	22,105 ± 45								
23,745	23,630 ± 160	AF	420	59.67	14.2	47.9	−18.6	14.0	3.5
X-2717-25	30,430 ± 300	HYP	380	41.83	11.0	42.5	−23.7	15.4	5.0

Conventional radiocarbon age is expressed in years BP^54^. PCode refers to pretreatment code; AF is ultrafiltered collagen and HYP denotes the extraction of hydroxyproline from hydrolysed bone collagen^12,13^. UF gelatin yield denotes the weight of ultrafiltered gelatin in milligrams. %Yld is the percent yield of extracted UF gelatin as a function of the starting weight of the bone analysed. %C is the carbon present in the combusted sample (gelatin or hydroxyproline). Stable isotope ratios δ^13^C and δ^15^N are expressed in per mil (‰) relative to vPDB and AIR, respectively, with a mass spectrometric precision of ± 0.2‰^55^. C/N denotes the atomic ratio of carbon to nitrogen and is acceptable if it ranges between 2.9–3.5 in the case of collagen, or ~5.0 in the case of hydroxyproline

### DNA extraction and mtDNA enrichment

DNA was extracted from 30.9, 27.0 and 33.1 mg of powder removed from the sampling spots B, C and D, respectively (Fig. 2)^26^. Aliquots of the extracts were converted to single-stranded libraries^27^ and enriched for human mtDNA fragments using a present-day human mtDNA probe set^28^. The enriched library molecules were sequenced from both ends. Overlapping paired-end reads were merged to reconstruct full-length molecules sequences, which were then mapped to the revised Cambridge reference sequence of the human mitochondrial genome (rCRS, GenBank acc. no. NC_0120920). After discarding sequences shorter than 35 base pairs (bp) and fusing sequences with identical start and end coordinates, 264,884 unique mtDNA fragments remained (Table 2).

**Table 2:** Characteristics of the mtDNA molecules isolated from the Salkhit skullcap

Library	#Unique mapped fragments (>=35 bp)	mtDNA coverage	Contamination estimate (%)	5’ C to T frequency (%)	Cond. 5’ C to T frequency (%)	3’ C to T frequency (%)	Cond. 3’ C to T frequency (%)	#deam. fragments	mtDNA coverage (deam. only)	Contamination estimate (%) (deam. only)
Salkhit - B	67,434	230.4	19.6	41.6	44.9	25.7	27.3	10,795	35.0	4.1
Salkhit - C	67,340	232.1	35.2	40.2	45.2	24.4	27.4	16,839	32.9	1.6
Salkhit - D	130,110	466.4	42.6	34.9	41.7	20.6	26.0	10,390	55.1	3.7
Negative control	64	0.2	–	14.3	0	7.1	0	4	0.01	–

The contamination proportion is estimated based on positions 10,364 and 14,578 (in the rCRS reference genome) diagnostic of the Salkhit individual

*Cond.* conditional, *deam.* deaminated

To assess whether or not molecules of ancient origin were present, we estimated the frequencies of cytosine (C) to thymine (T) mismatches along the sequences relative to the reference sequence. Such mismatches result from deamination of C residues in ancient DNA, particularly towards the ends of molecules^29^. The C to T mismatch frequencies ranged from 34.9% to 41.6% at the first (5’) position and from 20.6% to 25.7% at the last (3’) position of the sequences, respectively (Table 2) (Supplementary Fig. 1), with the library yielding the largest number of mitochondrial sequences exhibiting the lowest terminal C to T mismatch frequencies. The differences among the libraries suggest that, in addition to endogenous ancient DNA molecules, contaminating present-day human mtDNA molecules were extracted and incorporated in the libraries. This is further supported by the observation that DNA fragments that carry C to T mismatches at one end have higher frequencies of C to T mismatch at the other end than when all DNA fragments are considered (Table 2) (Supplementary Table 2)^30^. We conclude that the DNA libraries contain authentic ancient molecules carrying terminal C to T mismatches as well as present-day contaminating DNA molecules.

### Contamination and Salkhit mtDNA consensus sequence

To reduce the impact of cytosine deamination on the reconstruction of the mitochondrial genome, we masked T bases at the first and last three positions of each mtDNA fragment before aligning them to the rCRS reference human mitochondrial genome. The estimation of present-day human contamination in each library relied on two positions (pos. 10,364 and pos. 14,578 in the rCRS reference genome) where the Salkhit mtDNA genome sequence differs from a worldwide panel of 311 present-day human mtDNA genomes. The proportions of sequences covering both positions and not matching the Salkhit-specific state are 19.6%, 35.2% and 42.6% for the libraries made out the extracts B, C and D, respectively (Table 2).

Because of the high level of contamination of the specimen, we called the Salkhit mtDNA consensus using only the 38,024 mtDNA fragments carrying C to T mismatches to the human reference mtDNA in one of their three first or three last positions recovered from the three DNA libraries. This significantly lowers the amount of modern human contaminating sequences and reduces any potential bias for contaminating molecules resulting from the use of modern human capture baits and reference sequence for mapping. The consensus was called for 16,567 positions, out of 16,569, where at least 80% of the fragments supported the same base (Supplementary Fig. 2) with an average coverage of 123 DNA fragments per nucleotide position. One of the two missing positions was in a C-homopolymer stretch (pos. 295–320), which after visual inspection was manually corrected, while the other (pos. 16,093) is covered by 117 fragments from which 88 carry a C while 29 carry a T (Supplementary Fig. 2). This observation might reflect heteroplasmy and was left uncalled.

For the alignments of deaminated fragments only, we estimated the level of contamination to 4.1%, 1.6% and 3.7% for libraries made from extracts B, C and D, respectively (Table 2). Given the coverage of the genome, this level of contamination is not expected to affect the accuracy of the consensus sequence.

### MtDNA phylogeny and dating

**Fig. 5:**
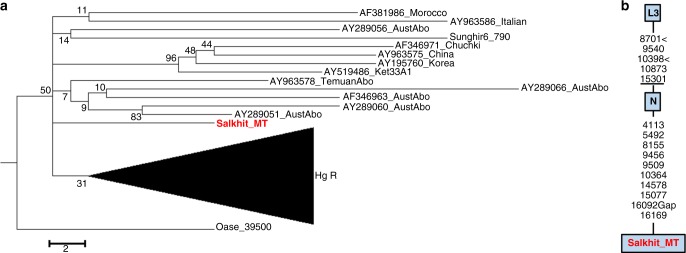
Maximum parsimony phylogeny relating the Salkhit mtDNA to ancient and present-day human mtDNAs. **a** Subtree of the N haplogroup (Hg) phylogeny structure extracted from a global tree including all the mtDNAs diversity found in ancient and present-day anatomically modern human (Supplementary Fig. 3). The tree was obtained in MEGA6 (30, 31) and is #1 out of 3 most parsimonious trees putting in a phylogenetic network 58 ancient and 55 present-day modern human mtDNA including the Salkhit sequence. The mtDNA sequence of a Neanderthal from Altai is used as outgroup. The percentage of replicate trees in which the associated taxa clustered together in the bootstrap test (1000 replicates) are shown next to the branches. **b** Diagnostic positions of the N haplogroup and positions of the substitutions accumulated in the Salkhit mtDNA since its divergence from the common ancestor of the N haplogroup, indicated along the branch, are obtained using mtPhyl (https://sites.google.com/site/mtphyl/home)

The Salkhit mtDNA lineage was assigned to the modern human macro-haplogroup N using HaploGrep2^31^ (based on mtDNA tree Build 17^32^). Haplogroup N and M are the two basal mtDNA haplogroups shared among all present-day non-Africans. While the mosaic of archaic-like and modern human-like morphological traits have made the assignment of the fossil to Pleistocene hominin groups difficult, we thus show that the mtDNA of the Salkhit individual is of modern human origin. However, the sequence does not carry any substitutions characteristic of known sub-haplogroups inside the haplogroup N. A maximum parsimony analysis (subtree Fig. 5) assigns the Salkhit mtDNA to an uncharacterized lineage which branches off the root of haplogroup N. It is therefore unlikely that the Salkhit mitochondrial lineage is directly ancestral to any present-day human mtDNA. Among ancient modern humans, only the mtDNA of the ~40,000-year-old Romanian *Oase 1* individual^33^ falls outside the known sub-lineages of N or M, suggesting the existence of more mtDNA diversity among early modern humans in Eurasia than among later and present-day Eurasian populations.

The maximum parsimony tree (Fig. 5) suggests that the Salkhit mtDNA accumulated 10 substitutions since its divergence from its closest relatives in the N haplogroup. Using a mtDNA mutation rate of 2.67 × 10^−8^ substitution per site per year^34^, we estimate that the Salkhit mtDNA diverged from the root of the N haplogroup 19,000 to 28,000 years (95% HPD) before the Salkhit individual lived. Considering the reported divergence time of the N haplogroup between 70 and 50 ka ago^35–37^, this estimate is compatible with the calibrated radiocarbon date.

We also estimated the age of the Salkhit specimen mtDNA using a Bayesian method implemented in BEAST^38^ and 58 mtDNA sequences from archaeologically dated specimens as calibration points (Supplementary Table 3). Applying a strict clock and the mutation rate above we obtain a date between 12,910 and 39,410 years BP (95% HPD) with a median of 26,600 years BP, supported by a Bayesian inference of phylogeny tree (Supplementary Fig. 4). This date is consistent with the calibrated radiocarbon date as well as with the molecular date inferred from the number of private substitutions accumulated in the Salkhit mtDNA since its divergence from the root of the N haplogroup.

### Conclusions

The morphological traits of the Salkhit skullcap have fueled the debate about the origins of the prehistoric humans of the region, where it is, to our knowledge, the only Pleistocene human fossil discovered. The complete mtDNA sequence reconstructed from the Salkhit skull falls within the variation of modern human mtDNAs, showing that the maternal lineage of the specimen is of modern human ancestry. The compound-specific radiocarbon date shows that the previous dates of 22 and 23 ka BP were too young. This is due to the presence of a silicon-based contaminant that could not be removed with other pretreatments. It is now clear that the Salkhit individual belongs within the EUP in Mongolia. Based on this new chronological evidence and its location, we hypothesize that the Salkhit individual, although found without any archaeological context, may have been familiar with the Levallois-like technology and blade production industry of the EUP found in Northern Mongolia and in Southern Siberia.

## Methods

### Radiocarbon dating

Two different methods were used to prepare the samples for AMS dating. First, samples were pre-treated following the routine procedure at the Oxford Radiocarbon Accelerator Unit (ORAU) comprising a decalcification, base wash, reacidification, gelatinisation and ultrafiltration (Coded ‘AF’ in the ORAU)^12^. The second sample taken from the skullcap was dated using the single amino acid radiocarbon dating method optimized at the ORAU^13^. This method involves separation of the underivatised amino acids from hydrolyzed bone collagen samples using preparative High Performance Liquid Chromatography (Prep-HPLC). The amino acid hydroxyproline is isolated by Prep-HPLC, combusted, graphitized and AMS dated. This pretreatment approach (Coded ‘HYP’ in the ORAU) is the most efficient technique to remove contaminants including, but not limited to, conservation materials (unless collagen-based glue has been applied). The %C, %N and atomic C/N ratio were measured using an automated carbon and nitrogen elemental analyzer (Carlo Erba EA1108) coupled with a continuous-flow isotope monitoring mass spectrometer (Europa Geo 20/20).

### DNA sampling, extraction and mtDNA sequence determination

After the removal of approximately 1 mm of surface material using a sterile dentistry drill, which was performed to reduce present-day human DNA contamination, the sampling for DNA analyses was carried out in areas B, C and D (Fig. 2). DNA was extracted from 30.9 mg, 33.1 mg and 27 mg, respectively, using a silica-based protocol optimized for the recovery of short molecules from ancient material^26^. An extraction blank was also carried through each step of DNA extraction. Ten microliters of each extract (of a total of 50 µL) were converted into a single-stranded DNA library as described elsewhere^27^ using an automated liquid handling platform^39^. One positive and one negative control were included during library preparation^40^. No uracil-DNA glycosylase treatment was performed. After amplification with a pair of primers carrying unique index sequences^41^ and quantification using a NanoDrop ND-1000 (NanoDrop Technologies) photospectrometer, 1 µg of each sample library and the blank library were enriched for mtDNA using present-day human mtDNA probes (in 1-bp tiling density) as described^28,34^. Before sequencing, libraries were amplified in a one-cycle-PCR using Herculase II Fusion DNA polymerase (Agilent)^42^ with primers IS5 and IS6^43^ to remove heteroduplices.

The libraries were pooled and sequenced on a MiSeq (Illumina Inc.) using a paired-end configuration of 76 + 7 cycles for each insert and index read^41^. Base calling was performed using Bustard (Illumina), and sequences that did not exactly match one of the expected index combinations were discarded. Adapter sequences were trimmed and overlapping paired-end reads merged using leeHom^44^ before mapping the sequences to the revised Cambridge Reference mitochondrial genome (rCRS, NC_0120920) using the Burrows-Wheeler Aligner (BWA)^45^ with parameters « -n 0.01 –o 2 –l 16500 »^46^. Mapped sequences with identical alignment start and end coordinates were collapsed using bam-rmdup (https://bitbucket.org/ustenzel/biohazard). Only overlap-merged sequences of at least 35 base pairs in length were retained for subsequent analyses.

After masking terminal C to T substitutions, a perl script was used to call the consensus mtDNA sequence (based on the «mpileup» command of SAMtools^47^) requiring a minimum coverage of 5 and a consensus support of at least 80% at each position.

### Phylogenetic analyses

The Salkhit mtDNA haplogroup was assigned using Haplogrep^31^ and mtPhyl5 (https://sites.google.com/site/mtphyl/home) based on the Phylotree database (mtDNA tree Build 17)^32^. The number of mutations that occurred in the Salkhit mtDNA since its divergence from the root of haplogroup N was inferred by maximum parsimony analysis using mtPhyl.

A multiple sequence alignment of the Salkhit mtDNA sequence to 115 complete mtDNAs of modern humans^18,34,36^ and one Neandertal mtDNA^48^ was generated using MAFFT^49^. We then estimated the molecular age of the Salkhit mtDNA based on its branch length in a Bayesian statistical framework^38^ as implemented in BEAST2^50^ using the dataset above. We used tip dates of zero for present-day mtDNA sequences and individual fossil dates intervals as priors for the ancient mtDNA sequences (Supplementary Table 3). As determined by jModelTest2, we performed the analyses using a GTR  +I+G model for nucleotide substitution with a gamma distributed rates among sites. A strict clock and an uncorrelated lognormal-distributed relaxed clock, both under a constant size population and a Bayesian skyline coalescent tree prior, were tested. To estimate the posterior distribution of each model, we performed two runs of the Markov Chain Monte Carlo (MCMC) algorithm implemented in the Beast software. Each MCMC run was performed with 50,000,000 iterations and sampled every 10,000 generations. After discarding 10% of the sampled iterations as burn-in, the output was analyzed with Tracer v1.5.0 (http://tree.bio.ed.ac.uk/software/tracer/). The effective sample sizes estimate supported the strict clock model over the relaxed clock model, while neither the constant size nor the Bayesian skyline coalescent models were rejected. The age of the Salkhit mtDNA was therefore estimated based on a strict clock model and a Bayesian skyline coalescent model.

### Pyrolysis gas chromatography coupled with mass spectrometry

The analyses were performed on an EGA/PY-3030D Micro Furnace Pyrolyzer (Frontier Lab, Japan) connected to a 6890 gas chromatograph equipped with a split/splitless injector. An HP 5MS column (30 m × 0.25 mm, film thickness 0.25 µm, Agilent Technologies, USA) coupled with a deactivated silica pre-column (2 m × 0.32 mm, Agilent Technologies, USA) was used for the chromatographic separation. The program of the oven temperature used for the chromatographic separation was 40 °C isothermal for 6 min followed by a linear ramp at 20 °C/min up to 310 °C. Analyses were performed under a constant flow of helium at 1 mL/min, with a split ratio 1:20. The gas chromatograph was coupled with a 5973 Mass Selective Detector (Agilent Technologies, USA). The MS transfer line temperature was 300 °C. The mass spectrometer was operated in EI positive mode (70 eV, scanning m/z 50-600). The MS ion source was kept at 230 °C and the MS quadrupole at 150 °C.

The two samples, 450 µg of bone powder from the Salkhit skull and 250 µg of an uncontaminated mammoth bone sample from Latton (UK), used as a background standard at the ORAU, were successively placed in a stainless-steel cup and inserted in the micro-furnace where they were pyrolysed at 600 °C for 20 sec.

### Reporting summary

Further information on experimental design is available in the Nature Research Reporting Summary linked to this article.

## Supplementary information

Supplementary Information

Reporting Summary

## Data Availability

The complete mitochondrial DNA sequence of the Salkhit individual is deposited in the European Nucleotide Archive (http://www.ebi.ac.uk/ena) under the accession number PRJEB29760. Other data that support the findings of this study are available from the corresponding authors on reasonable request. A reporting summary for this Article is available as a Supplementary Information file.
