# Supplementary material for: Compound-specific radiocarbon dating and mitochondrial DNA analysis of the Pleistocene hominin from Salkhit Mongolia
Source: Nat Commun. 2019 Jan 30;10:274. doi: 10.1038/s41467-018-08018-8 (PMC6353915; doi:10.1038/s41467-018-08018-8)
Supplement: Supplementary file 1 — Supplementary Information [file 41467_2018_8018_MOESM1_ESM.pdf]

Supplementary Information

Compound-specific radiocarbon dating and mitochondrial DNA analysis of the Pleistocene hominin from Salkhit Mongolia

Devièse et al.

This PDF includes:

- Supplementary Figures 1 to 4
- Supplementary Tables 1 to 3
- Supplementary References

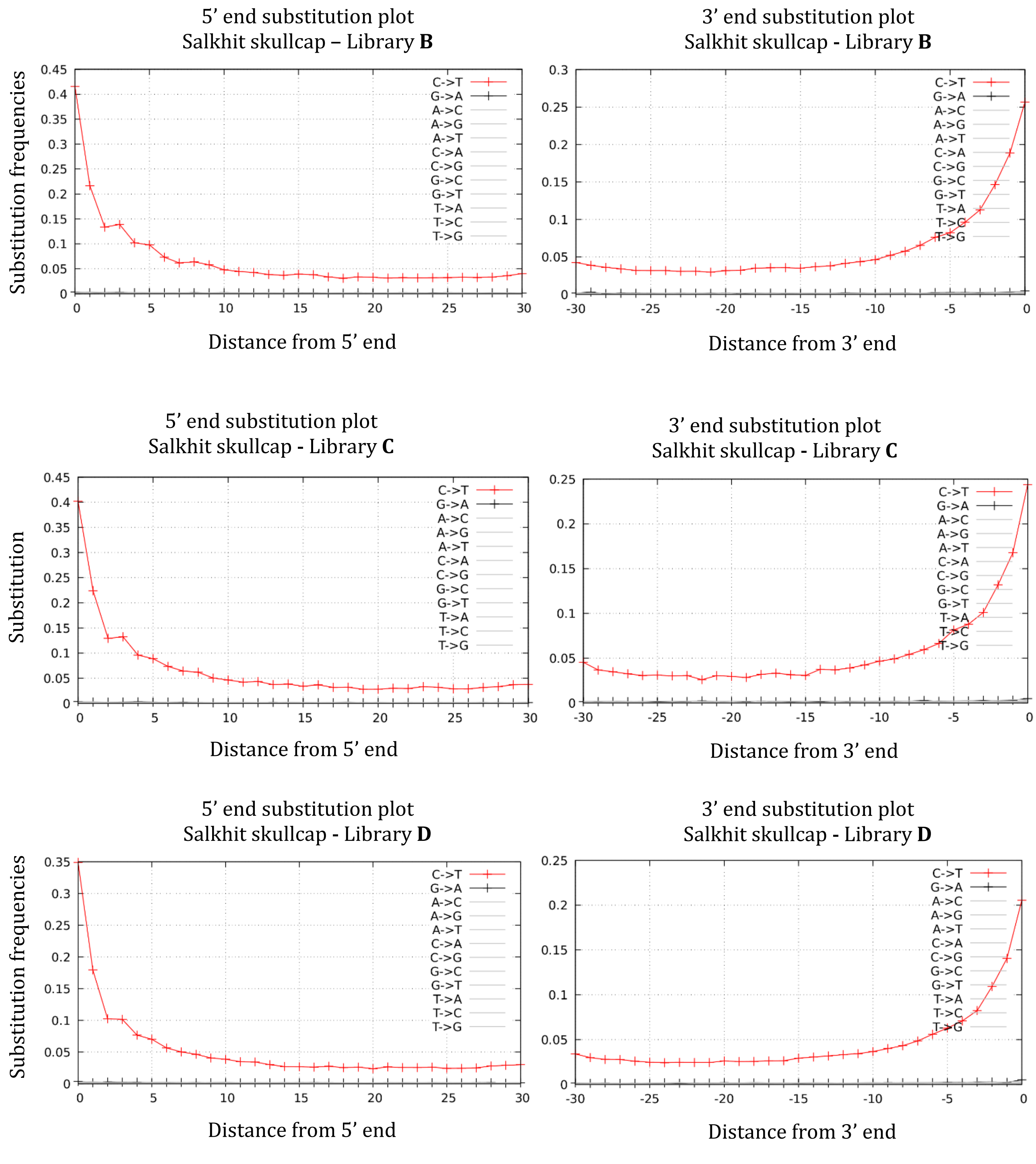

Supplementary Figure 1. Pattern of cytosine (c) to thymine (T) substitution in the three DNA libraries from Salkhit DNA extracts B, C and D. Plots of the frequencies of C in the reference mtDNA that occur as T among all fragments sequenced (red) are shown for the 30 terminal positions.

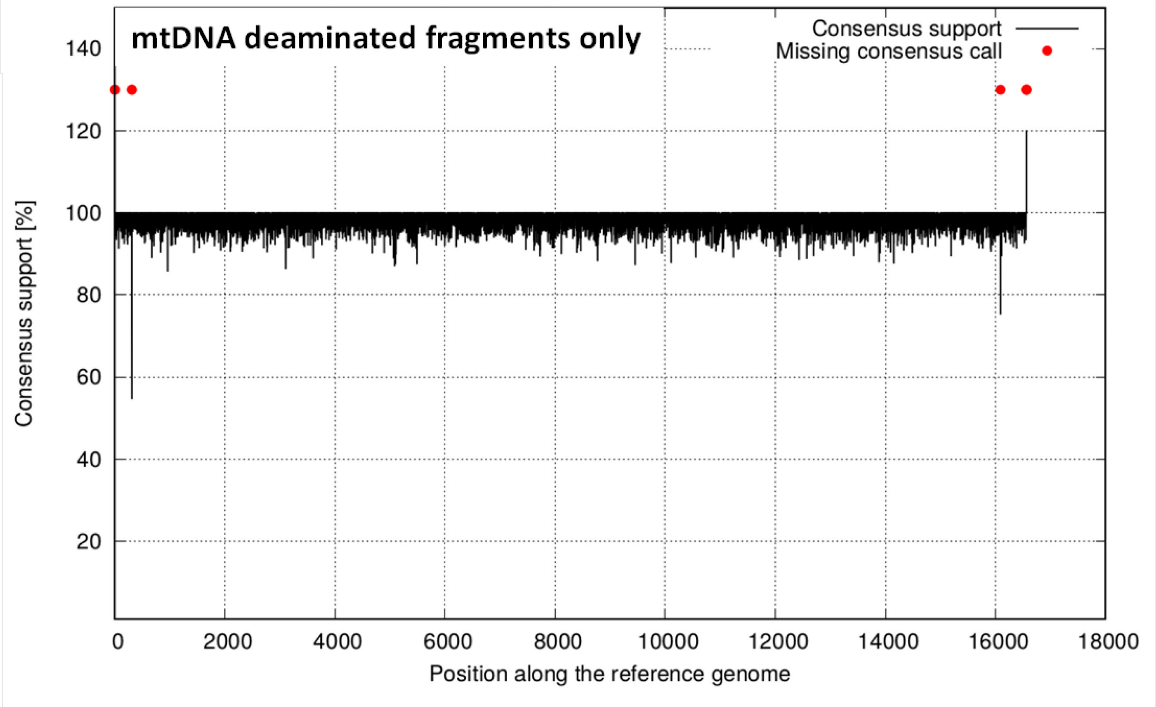

Supplementary Figure 2. Consensus support for each position of the mtDNA sequence of the Salkhit individual using DNA fragments showing evidence of cytosine deamination at least one of their three first or three last positions mapped to the rCRS reference mtDNA (top). In the former consensus mtDNA sequence 2 positions have less than 80% support (red dots).

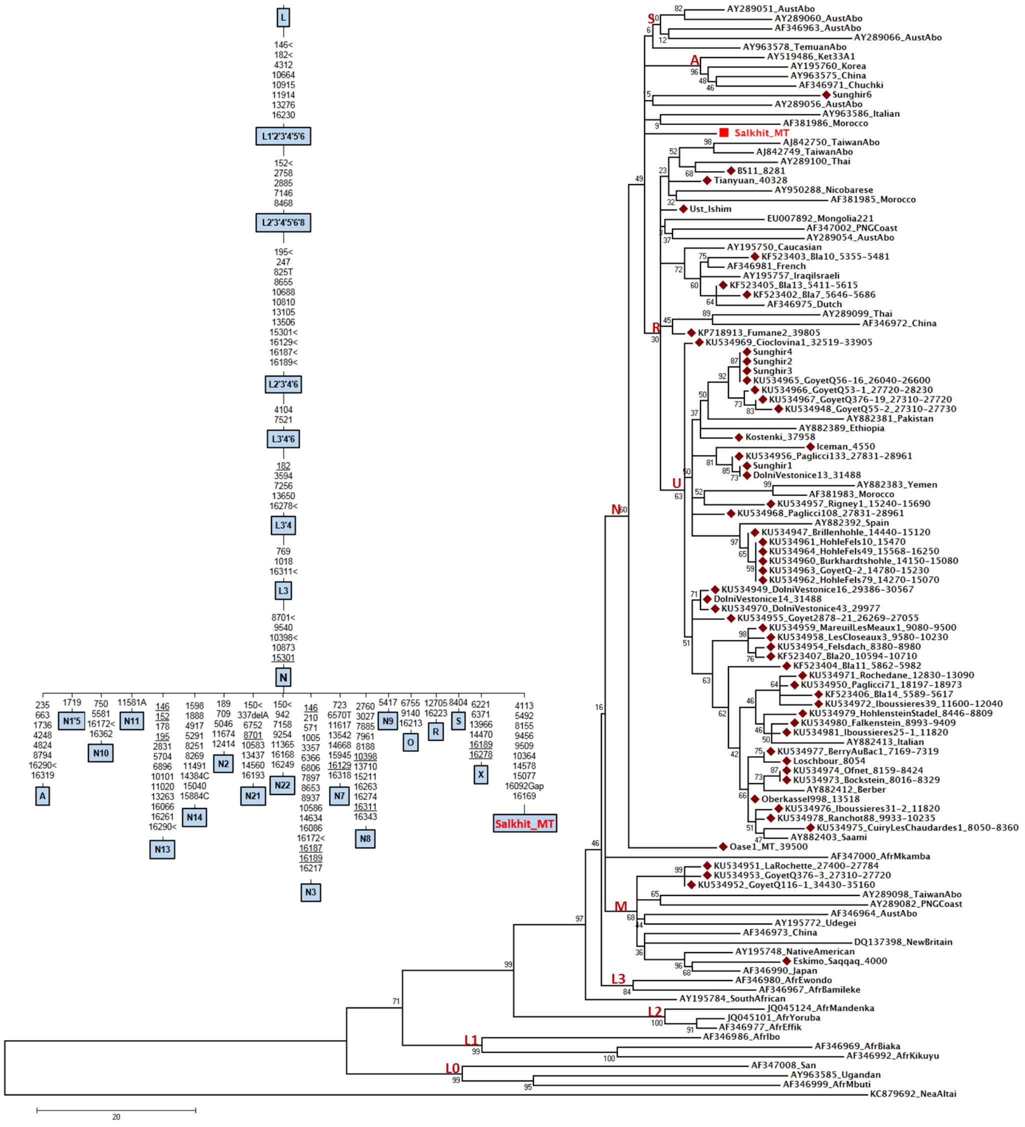

Supplementary Figure 3. Maximum Parsimony of ancient and present-day modern human mtDNA including the Salkhit sequence. The evolutionary history of the Salkhit mtDNA sequence was inferred using the Maximum Parsimony method. The subtree on the left was obtained using mtPhyl (https://sites.google.com/site/mtphyl/home) and shows the Salkhit mtDNA embranchment among the main sub-clades of the N haplogroup while categorizing the substitutions accumulated in the Salkhit mtDNA since it's divergence from the N-haplogroup clades common ancestor. The tree on the right was obtained in MEGA6 [1] and is #1 out of 3 most parsimonious trees (length = 1327) putting in a phylogenetic network 58 ancient (marked by a cardinal red diamond) and 55 present-day modern human mtDNA including the Salkhit sequence (marked by a red square). The mtDNA sequence of a Neanderthal from Altai is used as outgroup. The percentage of replicate trees in which the associated taxa clustered together in the bootstrap test (1000 replicates) are shown next to the branches. The tree was obtained using the Subtree-Pruning-Regrafting (SPR) algorithm and drawn to scale, with branch lengths in the units of the number of changes over the whole sequence calculated using the average pathway method. The nodes leading to the main mtDNA haplogroups are noted by the name of the corresponding haplogroup in red.

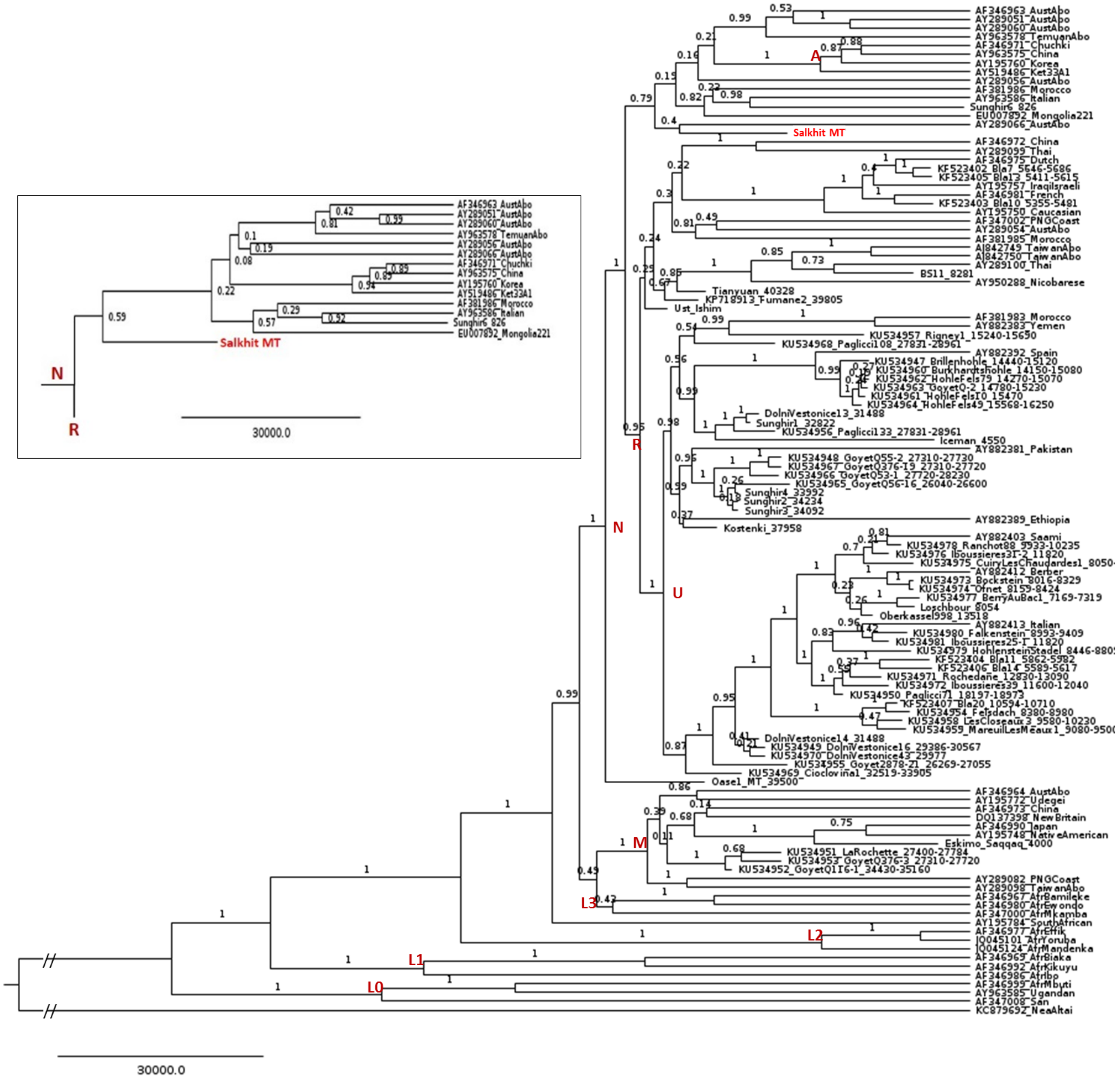

Supplementary Figure 4. Bayesian phylogenetic tree of the Salkhit mtDNA and ancient and present-day human mtDNAs. Several population models were tested and they differ in the branching structure among sub-clades inside the N haplogroup, including two distinct branching for the Salkhit mtDNA, both shown here. The full tree represent the best supported tree obtained using a strict molecular clock and Bayesian Skyline population size model, and the subtree a well less supported tree with a slightly different branch structure within the N haplogroup. The statistical supports of the nodes (Bayesian posterior probability) are indicated along the branch and the designations of the main haplogroups are given in red. The scale bar denotes the time in year.

Supplementary Table 1. Chronometric data of Mongolian Upper Paleolithic sites. The location of the sites is reported on Fig. 1.

Site / Phase	Location	Age (ka BP)	Reference
Tolbor 4 (Horizon 3)<br>Tolbor 4 (Horizon 4)<br>Tolbor 4 (Horizon 5)<br>Tolbor 4 (Horizon 5)<br>Tolbor 4 (Horizon 6)<br>Tolbor 4 (Horizon 6)	1	14547 ± 73 BP (AA-93139)<br>26700 ± 300 BP (AA-84135)<br>31210 ± 410 BP (AA-93140)<br>>41050 BP (AA-79326)<br>35230 ± 680 BP (AA-93141)<br>37400 ± 2600 BP (AA-79314)	[2]<br>[2]<br>[2]<br>[2-4]<br>[2]<br>[2-4]
Tolbor 15 (Horizon 3)<br>Tolbor 15 (Horizon 3)<br>Tolbor 15 (Horizon 4)<br>Tolbor 15 (Horizon 4)<br>Tolbor 15 (Horizon 5)<br>Tolbor 15 (Horizon 5)<br>Tolbor 15 (Horizon 7)<br>Tolbor 15 (Horizon 7)<br>Tolbor 15 (Horizon 7)<br>Tolbor 15 (Horizon 7)<br>Tolbor 15 (Horizon 7)<br>Tolbor 15 (Horizon 7)	1	14055 ± 80 BP (AA-84136)<br>14930 ± 70 BP (Beta-263742)<br>14680 ± 70 BP (Beta-263744)<br>14820 ± 70 BP (Beta-263745)<br>28460 ± 310 BP (AA-84137)<br>32200 ± 1400 BP (AA-93137)<br>26700 ± 300 BP (AA-84135)<br>29150 ± 20 BP (AA-84138)<br>33200 ± 1500 BP (AA-93137)<br>34010 ± 200 BP (MAMS-14934)<br>33470 ± 190 BP (MAMS-14935)<br>34340 ± 210 BP (MAMS-14937)	[2-5]<br>[2-4]<br>[2-4]<br>[2-4]<br>[2-4]<br>[2]<br>[3,4]<br>[2-4]<br>[2]<br>[2]<br>[2]<br>[2]
Tolbor 16 (Unit 4)<br>Tolbor 16 (Unit 7)<br>Tolbor 16 (Test pit (Pit 1) Unit 7)	1	15660 ± 40 BP (MAMS-14938)<br>33320 ± 180 BP (MAMS-14932)<br>>45400 BP (AA-93134)	[2]<br>[2]<br>[2]
Tolbor 17 (Test pit 2, Level 3)	1	29230 ± 930 BP (AA-93135)	[2]
Tolbor 21 (Test pit 1, Level 3)<br>Tolbor 21 (Test pit 1, Level 4)	1	39240 ± 360 BP (MAMS-14936)<br>44640 ± 690 BP (MAMS-14933)	[2]<br>[2]
Dörölj 1	2	29910 ± 310 BP (GifA-99560)<br>29540 ± 390 BP (GifA-99561)<br>31880 ± 800 BP (GifA-11664)<br>21820 ± 190 BP (GifA-102451)<br>22030 ± 180 BP (GifA-102453)	[2,6]<br>[2,3]<br>[2,3,6]<br>[2,3]<br>[2,3]
Orkhon 1 (Unit 1)	3	34400 ± 600 BP	[6]
Orkhon 7 (Excavation Unit 1, Horizon 2)<br>Orkhon 7 (Excavation Unit 3, Horizon 2)<br>Orkhon 7 (Excavation Unit 3, Horizon 2)<br>Orkhon 7 (Layer 5)<br>Orkhon 7 (Layer 7)<br>Orkhon	3	23595 ± 155 BP (SOAN-2883)<br>24400 ± 1100 BP *<br>25500 ± 1400 BP *<br>25,5 ky BP<br>35265 ± 500 BP<br>33785 ± 300 BP (SOAN-2885)	[4]<br>[4]<br>[4]<br>[6]<br>[6]<br>[4]
Chikhen Agui	4	27432 ± 872 BP (AA-26580)	[3,4,7]
Chikhen 2 (Horizon 2.5)	4	30550 ± 410 BP (AA-31870)	[3,4,7]
Tsagaan Agui (Stratum 3)	5	30942 ± 478 BP (AA-26589)<br>33840 ± 640 BP (AA-23158)	[7]<br>[7]
Rashaan Khad	6	39,100 ± 1,000 BP (OxA-34324)	[8]
Salkhit	7	30400 ± 300 BP (X-2717-25)	this paper

* dates generated by non-equilibrium uranium methods (Pa-231 and Th-230)

Supplementary Table 2. Characteristics of the mtDNA enriched libraries generated from the Salkhit skullcap. Seq = sequences, Cond = conditional deamination frequency of putative ancient DNA molecules in the libraries, deam = deaminated. The contamination proportion is estimated based on positions 10,364 and 14,578 (in the rCRS reference genome) specific of the Salkhit individual.

Library	**# Total seq. generated**	**#Fragment >= 35 bp**	**#Unique mapped fragments (>= 35 bp)**	**Average duplication rate**	**Average fragment size (bp)**	**5' C to T frequency (%)**	**Cond. 5' C to T frequency (%)**	**3' C to T frequency (%)**	**Cond. 3' C to T frequency (%)**	**#deam. fragments**	**Average fragment size (bp)**
Salkhit - B	1,478,545	1,100,636	67,434	10.4	56.6	41.6	44.9	25.7	27.3	10,795	53.7
Salkhit - C	1,609,955	1,193,323	67,340	11.4	57.1	40.2	45.2	24.4	27.4	16,839	52.4
Salkhit - D	1,560,580	1,183,491	130,110	5.8	59.4	34.9	41.7	20.6	26.0	10,390	54.2
Negative control	183,528	56,303	64	810.2	56.9	14.3	0	7.1	0	4	56.5

Supplementary Table 3. Ancient specimens used for the mtDNA phylogeny. For each sample the GenBank accession number, the country of origin, and the uncalibrated and calibrated (using the IntCal13 calibration curve of Reimer et al.[13]) radiocarbon date are reported. The specimens marked by * have not been directly dated.

Sample	GenBank Accession Number	origin (Country)	Uncalibrated (BP)		Calibrated (IntCal13) (BP)		
			ages	±	upper	lower	mean
**BerryAubac1**	KU534977	France	6325	35	7320	7168	7244
**BLA10**	KF523403	Germany	4590	25	5445	5085	5265
**BLA11**	KF523404	Germany	5145	30	5987	5759	5873
**BLA13**	KF523405	Germany	4730	25	5583	5327	5455
**BLA14**	KF523406	Germany	4835	30	5644	5478	5561
**BLA20**	KF523407	Germany	9370	45	10713	10441	10577
**BLA7**	KF523402	Germany	4860	30	5653	5488	5571
**Bockstein***	KU534973	Germany	7350	70	8331	8016	8174
**Brillenhohle**	KU534947	Germany	12470	65	15034	14245	14640
**BS11**	KC521454	China	7368	34	8316	8048	8182
**Burkhardtshohle**	KU534960	Germany	12450	110	15080	14153	14617
**Cioclovina1**	KU534969	Romania	28510	170	33085	31788	32437
**CuiryLesChaudardes1**	KU534975	France	7400	60	8363	8048	8206
**DolniVestonice13**	KC521459	Czech Republic	26640	110	31070	30666	30868
**DolniVestonice14**	KC521458	Czech Republic	26640	110	31070	30666	30868
**DolniVestonice16***	KU534949	Czech Republic	25740	210	30575	29395	29985
**DolniVestonice43***	KU534970	Czech Republic	25740	210	30575	29395	29985
**Eskimo_Saqqaq**	EU725621	Greenland	4044	31	4783	4423	4603
**Falkenstein**	KU534980	Germany	8185	80	9407	8994	9201
**Felsdach***	KU534954	Germany	7770	120	8979	8380	8680
**Fumane2**	KP718913	Italy	33140	460	38523	36249	37386
**Goyet2878-21**	KU534955	Belgium	22360	110	27058	26272	26665
**GoyetQ116-1-3**	KU534952	Belgium	30880	170	35166	34436	34801
**GoyetQ-2**	KU534963	Belgium	12650	50	15232	14778	15005
**GoyetQ376-19**	KU534967	Belgium	23260	110	27718	27313	27516
**GoyetQ376-3**	KU534953	Belgium	29370	180	33941	33153	33547
**GoyetQ53-1**	KU534966	Belgium	23920	100	28231	27719	27975
**GoyetQ55-2**	KU534948	Belgium	23270	120	27731	27311	27521
**GoyetQ56-16**	KU534965	Belgium	22100	100	26602	26041	26322
**HohleFels10***	KU534961	Germany	12770	220	15873	14260	15067
**HohleFels49***	KU534964	Germany	12770	220	15873	14260	15067
**HohleFels79**	KU534962	Germany	12490	70	15071	14267	14669
**HohlensteinStadel**	KU534979	Germany	7835	80	8978	8448	8713
**Iboussieres25-1***	KU534981	France	10140	50	12040	11411	11726

**Iboussieres31-2***	KU534976	France	10140	50	12040	11411	11726
**Iboussieres39**	KU534972	France	10140	50	12040	11411	11726
**Iceman**	Ermini & al. 2008 [9]	Italy	4550	19	5315	5067	5191
**Kostenki**	FN600416	Russia	33250	500	38684	36262	37473
**LaRochette**	KU534951	France	23630	130	27962	27516	27739
**LesCloseaux3**	KU534958	France	8870	130	10231	9564	9898
**Loschbour**	KC521455	Luxembourg	7205	50	8160	7948	8054
**MareuilLesMeaux1**	KU534959	France	8320	90	9496	9034	9265
**Oase**	Fu & al. 2014 [10]	Romania	34950	990	41761	37311	39536
**Oberkassel998**	KC521457	Germany	12180	100	14501	13758	14130
**Ofnet***	KU534974	Germany	7480	80	8425	8065	8245
**Paglicci108***	KU534968	Italy	23470	370	28430	27070	27750
**Paglicci133***	KU534956	Italy	28100	400	33103	31210	32157
**Paglicci71***	KU534950	Italy	15460	220	19245	18220	18733
**Ranchot88**	KU534978	France	8985	40	10236	9933	10085
**Rigney1**	KU534957	France	12930	55	15691	15240	15466
**Rochedane**	KU534971	France	11120	50	13089	12830	12960
**Sunghir1**	Sikora & al. 2017 [11]	Russia	28890	430	33875	31770	32823
**Sunghir2**	Sikora & al. 2017 [11]	Russia	30100	550	35283	33185	34234
**Sunghir3**	Sikora & al. 2017 [11]	Russia	30000	550	35154	33031	34093
**Sunghir4**	Sikora & al. 2017 [11]	Russia	29820	280	34485	33499	33992
**Sunghir6**	Sikora & al. 2017 [11]	Russia	884	23	905	732	819
**Tianyuan**	KC417443	China	34430	510	40254	37761	39008
**Ust_Ishim**	Fu & al. 2015 [12]	Russia	41400	1400	48313	42793	45553

Supplementary References

1 Tamura, K., Stecher, G., Peterson, D., Filipski, A. & Kumar, S. MEGA6: Molecular Evolutionary Genetics Analysis Version 6.0. *Molecular Biology and Evolution* **30**, 2725-2729, doi:https://doi.org/10.1093/molbev/mst197 (2013).

2 Zwyns, N. *et al.* The open-air site of Tolbor 16 (Northern Mongolia): Preliminary results and perspectives. *Quaternary International* **347**, 53-65, doi:https://doi.org/10.1016/j.quaint.2014.05.043 (2014).

3 Gladyshev, S. A., Olsen, J. W., Tabarev, A. V. & Jull, A. J. T. The Upper Paleolithic of Mongolia: Recent finds and new perspectives. *Quaternary International* **281**, 36-46, doi:https://doi.org/10.1016/j.quaint.2012.01.032 (2012).

4 Gladyshev, S. A., Olsen, J. W., Tabarev, A. V. & Kuzmin, Y. V. Chronology and Periodization of Upper Paleolithic Sites in Mongolia. *Archaeology, Ethnology and Anthropology of Eurasia* **38**, 33-40, doi:https://doi.org/10.1016/j.aeae.2010.10.003 (2010).

5 Zwyns, N., Gladyshev, S., Tabarev, A. & Gunchinsuren, B. in *Encyclopedia of Global Archaeology* (ed Claire Smith) 5025-5032 (Springer New York, 2014).

6 Jaubert, J. *et al.* in *Le Paléolithique supérieur / The Upper Paleolithic. Sessions générales et posters / General Sessions and Posters, Actes du XIVe Congrès UISPP, Université de Liège, 2-8 sept. 2001 BAR International Series, 1240* (ed Section Secrétariat général du Congrès) 225-241. (Archaeopress, 2004).

7 Derevianko, A. P., Markin, S. V., Gladyshev, S. A. & Olsen, J. W. The Early Upper Paleolithic of the Gobi Altai Region in Mongolia Based on Materials from the Chikhen-2 Site. *Archaeology, Ethnology and Anthropology of Eurasia* **43**, 17-41, doi:https://doi.org/10.1016/j.aeae.2015.11.004 (2015).

8 Yi, S. Rashaan Khad - An Early Blade Industry in Eastern Mongolia. *Journal of the Korean Palaeolithic Society* **28**, 105-129 (2013).

9 Ermini, L. *et al.* Complete Mitochondrial Genome Sequence of the Tyrolean Iceman. *Current Biology* **18**, 1687-1693, doi:https://doi.org/10.1016/j.cub.2008.09.028 (2008).

10 Fu, Q. *et al.* Genome sequence of a 45,000-year-old modern human from western Siberia. *Nature* **514**, 445, doi:10.1038/nature13810 (2014).

11 Sikora, M. *et al.* Ancient genomes show social and reproductive behavior of early Upper Paleolithic foragers. *Science* **358**, 659-662, doi:10.1126/science.aao1807 (2017).

12 Fu, Q. *et al.* An early modern human from Romania with a recent Neanderthal ancestor. *Nature* **524**, 216-219, doi:10.1038/nature14558 (2015).

13 Reimer, P. J. *et al.* IntCal13 and Marine13 Radiocarbon Age Calibration Curves 0–50,000 Years cal BP. *Radiocarbon* **55**, 1869-1887, doi:https://doi.org/10.2458/azu_js_rc.55.16947 (2013).
