# Supplementary material for: Compound-specific radiocarbon dating and mitochondrial DNA analysis of the Pleistocene hominin from Salkhit Mongolia
Source: Nat Commun. 2019 Jan 30;10:274. doi: 10.1038/s41467-018-08018-8 (PMC6353915; doi:10.1038/s41467-018-08018-8)
Supplement: Supplementary file 2 — Reporting Summary [file 41467_2018_8018_MOESM2_ESM.pdf]

nature research

Corresponding author(s): Thibaut Devièse, Diyendo Massilani

Reporting Summary

Nature Research wishes to improve the reproducibility of the work that we publish. This form provides structure for consistency and transparency in reporting. For further information on Nature Research policies, see Authors & Referees and the Editorial Policy Checklist.

Statistical parameters

When statistical analyses are reported, confirm that the following items are present in the relevant location (e.g. figure legend, table legend, main text, or Methods section).

n/a	Confirmed	
☒	☐	The exact sample size (*n*) for each experimental group/condition, given as a discrete number and unit of measurement
☒	☐	An indication of whether measurements were taken from distinct samples or whether the same sample was measured repeatedly
☒	☐	The statistical test(s) used AND whether they are one- or two-sided<br>*Only common tests should be described solely by name; describe more complex techniques in the Methods section.*
☒	☐	A description of all covariates tested
☒	☐	A description of any assumptions or corrections, such as tests of normality and adjustment for multiple comparisons
☒	☐	A full description of the statistics including central tendency (e.g. means) or other basic estimates (e.g. regression coefficient) AND variation (e.g. standard deviation) or associated estimates of uncertainty (e.g. confidence intervals)
☒	☐	For null hypothesis testing, the test statistic (e.g. *F*, *t*, *r*) with confidence intervals, effect sizes, degrees of freedom and *P* value noted<br>*Give P values as exact values whenever suitable.*
☒	☐	For Bayesian analysis, information on the choice of priors and Markov chain Monte Carlo settings
☒	☐	For hierarchical and complex designs, identification of the appropriate level for tests and full reporting of outcomes
☒	☐	Estimates of effect sizes (e.g. Cohen's *d*, Pearson's *r*), indicating how they were calculated
☒	☐	Clearly defined error bars<br>*State explicitly what error bars represent (e.g. SD, SE, CI)*

Our web collection on statistics for biologists may be useful.

Software and code

Policy information about availability of computer code

Data collection	All stable isotope data has been produced using the Sercon Callisto software.
Data analysis	The C14 date was calibrated using OxCal 4.3.

For manuscripts utilizing custom algorithms or software that are central to the research but not yet described in published literature, software must be made available to editors/reviewers upon request. We strongly encourage code deposition in a community repository (e.g. GitHub). See the Nature Research guidelines for submitting code & software for further information.

Data

Policy information about availability of data

All manuscripts must include a data availability statement. This statement should provide the following information, where applicable:

- Accession codes, unique identifiers, or web links for publicly available datasets
- A list of figures that have associated raw data
- A description of any restrictions on data availability

The data that support the findings of this study are available from the corresponding authors on reasonable request.

Field-specific reporting

Please select the best fit for your research. If you are not sure, read the appropriate sections before making your selection.

☐ Life sciences ☐ Behavioural & social sciences ☒ Ecological, evolutionary & environmental sciences

For a reference copy of the document with all sections, see nature.com/authors/policies/ReportingSummary-flat.pdf

Ecological, evolutionary & environmental sciences study design

All studies must disclose on these points even when the disclosure is negative.

Study description	We present the results of both chronometric and genetic analyses of the Salkhit specimen. This fossil dates to approximately 34 - 35 thousand years ago and its mitochondrial genome, shows that it falls within modern human haplogroup N found across Eurasia.
Research sample	The skullcap analysed in this study was discovered in 2006 during mining operations in the Salkhit Valley of the Norovlin county in the Khentii province, eastern Mongolia (48°16′17.9″ N and 112°21′37.9″ E). It is, so far, the only Pleistocene human fossil found in the country.
Sampling strategy	The specimen was sampled for radiocarbon and genetic analyses in four locations in the internal part of the skull at the posterior tip next to an area that had been sampled prior to this study.
Data collection	Radiocarbon data was recorded at the Oxford Radiocarbon Accelerator Unit (ORAU, UK). Genetic data was recorded at the Max-Planck-Institute for Evolutionary Anthropology (Leipzig, DE)
Timing and spatial scale	*Indicate the start and stop dates of data collection, noting the frequency and periodicity of sampling and providing a rationale for these choices. If there is a gap between collection periods, state the dates for each sample cohort. Specify the spatial scale from which the data are taken*
Data exclusions	No data were excluded.
Reproducibility	Standards were analysed at the same time as the archaeological specimen to confirm that there was no anamaly in the procedure.
Randomization	There was only one specimen found. All analyses have been perfomed on the same specimen.
Blinding	No assumption was made in the analysis of the data generated.

Did the study involve field work? ☐ Yes ☒ No

Reporting for specific materials, systems and methods

Materials & experimental systems

n/a	Involved in the study
☒	☐ Unique biological materials
☒	☐ Antibodies
☒	☐ Eukaryotic cell lines
☐	☒ Palaeontology
☒	☐ Animals and other organisms
☒	☐ Human research participants

Methods

n/a	Involved in the study
☒	☐ ChIP-seq
☒	☐ Flow cytometry
☒	☐ MRI-based neuroimaging

Palaeontology

Specimen provenance	The skullcap analysed in this study was discovered in 2006 during mining operations in the Salkhit Valley of the Norovlin county in the Khentii province, eastern Mongolia (48°16′17.9″ N and 112°21′37.9″ E).
Specimen deposition	Since its discovery in 2006, the Salkhit fossil (Reference number: 2006-4) has been kept at the Institute of History and Archaeology (formerly, Institute of Archaeology), Mongolian Academy of Sciences, in Ulaanbaatar.
Dating methods	Two different methods were used to prepare the samples for AMS dating. First, samples were pre-treated following the routine procedure at the Oxford Radiocarbon Accelerator Unit (ORAU) comprising a decalcification, base wash, reacidification,

gelatinisation and ultrafiltration (Coded ‘AF’ in the ORAU). The second sample taken from the skullcap was dated using the single amino acid radiocarbon dating method optimized at the ORAU (Coded 'HYP'). The samples (collagen or hydroxyproline) were then combusted, graphitized and AMS dated.
Technical details of the pretreatment methods can be found in:
Brock, F., Higham, T., Ditchfield, P. & Bronk Ramsey, C. Current Pretreatment Methods for AMS Radiocarbon Dating at the Oxford Radiocarbon Accelerator Unit (ORAU). Radiocarbon 52, 103-112 (2010).
Devièse, T., Comeskey, D., McCullagh, J., Bronk Ramsey, C. & Higham, T. New protocol for compound specific radiocarbon analysis of archaeological bones. Rapid Communications in Mass Spectrometry 32, 373–379, doi:10.1002/rcm.8047 (2018).
The date presented in this article was calibrated using OxCal 4.3 and the INTCAL13 calibration curve.

☒ Tick this box to confirm that the raw and calibrated dates are available in the paper or in Supplementary Information.
